# Crossbar Nanoscale HfO_2_-Based Electronic Synapses

**DOI:** 10.1186/s11671-016-1360-6

**Published:** 2016-03-15

**Authors:** Yury Matveyev, Roman Kirtaev, Alena Fetisova, Sergey Zakharchenko, Dmitry Negrov, Andrey Zenkevich

**Affiliations:** Moscow Institute of Physics and Technology, Dolgoprudny, 141700 Russia

**Keywords:** Memristor, Crossbar, Electronic synapse, STDP, Resistive switching, HfO_2_, 85.50.-n, 81.07.-b, 84.35.+i

## Abstract

Crossbar resistive switching devices down to 40 × 40 nm^2^ in size comprising 3-nm-thick HfO_2_ layers are forming-free and exhibit up to 10^5^ switching cycles. Four-nanometer-thick devices display the ability of gradual switching in both directions, thus emulating long-term potentiation/depression properties akin to biological synapses. Both forming-free and gradual switching properties are modeled in terms of oxygen vacancy generation in an ultrathin HfO_2_ layer. By applying the voltage pulses to the opposite electrodes of nanodevices with the shape emulating spikes in biological neurons, spike-timing-dependent plasticity functionality is demonstrated. Thus, the fabricated memristors in crossbar geometry are promising candidates for hardware implementation of hybrid CMOS-neuron/memristor-synapse neural networks.

## Background

The processing of information following the classical von Neumann digital computing paradigms is known to be less efficient compared to their biological counterparts, when dealing with ill-posed problems and noisy data, such as image and voice recognition, pattern classification, navigation, etc. Though current computing technologies have reached the speed and computational power that allows them to simulate parts of animal brains and behavior, the energy required by these systems grows exponentially with the increasing hierarchy of animal intelligence. The reason is that the biological brain is configured differently and particularly features an extremely high level of connectivity between neurons, full integration of logic and memory functionality in the same components, and packaging in a compact 3D network. Such architecture results in the highly parallel operation, energy efficiency, adaptiveness, and self-learning of these networks [1]. The obvious advantages of a living brain have been motivating the development of artificial neural networks, which attempt to mimic the architecture of biological systems. Previously, the mainstream approach was focused on the software implementation of such networks utilizing classical von Neumann computers with separate memory and logic units. However, such approach restricts the efficiency of computation once it comes to difficult tasks such as pattern recognition and navigation. Therefore, in order to create an efficient artificial neural network, one needs to find the solution for a hardware implementation. Until recently, the “bottleneck” of the latter approach has been the lack of a compact device emulating the functionality of biological synapses.

Meanwhile, over the last decade, a broad set of reversible electrical resistance-switching phenomena in thin-film devices called memristors have attracted renewed attention as a functional basis for the alternative non-volatile memory technologies [1, 2]. Memristors offer several advantages, such as the scalability down to a few nanometers in size [3] and ultralow energy consumption [4], as well as the possibility of integration in high-dense matrices in the so-called crossbar geometry [3–6]. Further, such memristor matrices can be integrated with сomplementary metal-oxide-semiconductor (CMOS) technology, which paves the way for the design of reconfigurable logic devices [6].

By careful optimization of the applied electrical pulses, one can stabilize several intermediate resistance states in a memristor and eventually achieve an almost continuous spectrum of resistance states [7]. It has been previously demonstrated that memristors can display the functional properties of biological synapses, such as long-term potentiation and depression [8, 9], pair-pulse fluctuation [10], and spike-timing-dependent plasticity (STDP) [11]. It is therefore concluded that memristors can be viewed as “electronic synapses,” which provide an opportunity to build hybrid artificial neuromorphic computing systems, where memristor-based analog and CMOS-based digital logic parts are integrated in one device, thus combining the benefits from both technologies [12].

Among the variety of the previously investigated material systems, HfO_2_-based resistive switching devices are of special interest since this material has been integrated into the modern CMOS technology. Consequently, HfO_2_-based devices have been extensively studied in the context of the non-volatile memory applications [13–17], including several works demonstrating their functionality in crossbar geometry [18, 19]. Moreover, in several works, the synaptic properties on the stand-alone HfO_2_-based devices have been demonstrated [20]. The peculiar property of HfO_2_-based memristors is that while they exhibit gradual transition from low to high resistance states [9, 21], the reverse transition is usually abrupt and requires a compliance current to be set.

In this work, we report on TiN/HfO_2_/Pt devices scaled down to the lateral size 40 × 40 nm^2^ and integrated in crossbar geometry, which demonstrates the ability of gradual resistance switching in both directions. We explain the forming-free and gradual switching process by interface-limited trap-assisted tunneling mechanism previously adopted for HfO_2_-based devices. We emulate the synaptic functionalities such as long-term potentiation and depression. Furthermore, by applying the voltage pulses with the shape of real biological spikes, our devices demonstrate the spike-timing-dependent plasticity functionality. The emulated synaptic properties indicate that these devices can be used in hybrid neuromorphic computational systems.

## Methods

**Fig. 1:**
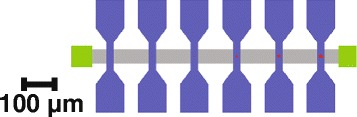
Layout of a memristor crossbar: *gray*—bottom electrode, *blue*—top electrode, *red*—memristor areas

The devices were formed on 2-in. Si(001) substrates with a 200-nm-thick SiO_2_ layer grown by plasma-enhanced chemical vapor deposition technique. In order to assess functional properties of individual devices with different lateral sizes in a crossbar, a 1 × 12 crossbar geometry was applied (Fig. 1). For this, a maskless optical lithography (Heidelberg Instruments μPG101) with the resolution ~1 μm was combined with an e-beam lithography (Crestec CABL 9000C) with the minimal line width in resist ~10 nm.

**Fig. 2:**
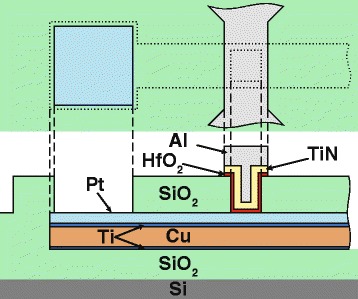
Schematic cross section of the fabricated memristor devices

The bottom electrode comprising 40-nm-thick Pt layer was deposited by e-beam evaporation on top of magnetron sputtered Ti(5 nm)/Cu(75 nm)/Ti(5 nm) layers and further patterned to the shape of a 800 × 50 μm^2^ beam with two 100 × 100 μm^2^ contact pads. To avoid a peculiar plasma-chemical and wet etching process for Pt, the bottom electrode was formed by a lift-off process. Following the formation of the bottom electrode, the sample was covered with 100-nm-thick SiO_2_, containing 12 windows with lateral sizes from 1.25 × 1.25 μm^2^ down to 40 × 40 nm^2^, produced by the e-beam lithography. At the next step, a functional HfO_2_ layer with different thickness in the range from 3 to 5 nm was grown by atomic layer deposition (ALD) in Sunale R-100 Picosun OY reactor at *T* = 240 °C utilizing Hf[N(CH_3_)(C_2_H_5_)]_4_ and H_2_O as the precursor for Hf and O, respectively. Finally, TiN top electrode 100 nm in thickness capped with a 100-nm-thick Al was deposited by magnetron sputtering and patterned by maskless laser lithography. The detailed description of the developed fabrication procedure is given in [22]. The schematic cross section of the formed devices is shown in Fig. 2.

## Results and Discussion

The electrical measurements were performed at room temperature using Cascade Summit 1100 probe station coupled with Agilent semiconductor device analyzer B1500A containing two source-measure units and two pulse generator units connected via a selector. During measurements, the common bottom electrode was grounded and the bias was applied to the top electrode.

**Fig. 3:**
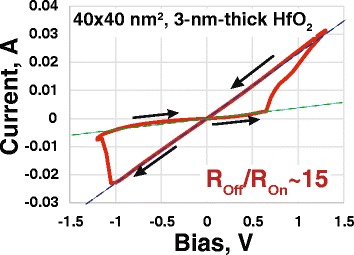
Typical *I*-*V* curve taken from 40 × 40 nm^2^ memristive crossbar device with a 3-nm-thick HfO_2_ layer

**Fig. 4:**
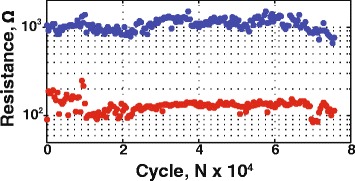
Endurance test on a 40 × 40 nm^2^ 3-nm-thick HfO_2_ device yielding 10^5^ switching cycles

**Fig. 5:**
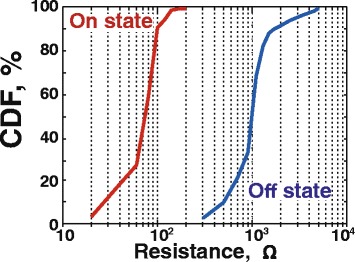
Cumulative distribution of the resistance in low (*On*) and high (*Off*) states for 100 cycles of 33 randomly selected structures from a 3-nm-thick HfO_2_ sample

In order to initiate the reversible resistive switching effect, as grown devices were subjected to the electrical forming by positive bias sweep with the current compliance of *I*_cc_ = 1 mA. Lower compliance current level during the forming process did not result in the higher resistance or smaller operation current. This is believed to be the consequence of limited reaction time of source-measurement units. After the forming procedure, the devices exhibit reversible switching in direct current (DC) *I*-*V* regime without any current compliance settings (Fig. 3). The endurance of the smallest (40 × 40 nm^2^) memristors in one-pulse switching mode (trapezoidal form *τ*_*FWHM*_ = 2 μs long pulse with 1 μs long tails) is ~7 × 10^4^ cycles (see Fig. 4). The cumulative probability of *R*_On_ and *R*_Off_ values for 100 cycles of 35 randomly selected structures for 3-nm-thick devices is presented in Fig. 5, demonstrating an acceptable level of uniformity (off-state of all devices are separated from on-states by a window). The typical ranges of the “SET” (“RESET”) voltages and resistance values for the devices with different oxide layer thickness are collected in the Table 1. One can see that most of the device properties are in the same range and do not depend on thickness. The only exception is the forming voltage, which obviously decreases for thinner structures. Moreover, for 3-nm-thick devices, the forming voltage decreases to the levels, where it partially “overlaps” the SET voltage. This is the criterion for the forming-free devices, which is beneficial in terms of their use in crossbar topologies.

**Table 1:** Typical values of the forming/switching voltage and SET/RESET resistance for memristor devices with different HfO_2_ layer thickness

*d*_HfO2_, nm	Forming voltage, V	*U*_Set_, V	*U*_Reset_, V	*R*_HRS_, Ω	*R*_LRS_, Ω
3	1 ÷ 2.3	0.7 ÷ 1.2	−1 ÷ −1.3	200 ÷ 6000	30 ÷ 200
4	1.3 ÷ 2.8	0.7 ÷ 1	−1 ÷ −1.3	200 ÷ 5000	35 ÷ 200
5	2.4 ÷ 3.8	0.7 ÷ 1	−0.95 ÷ −1.3	400 ÷ 5000	50 ÷ 400

**Fig. 6:**
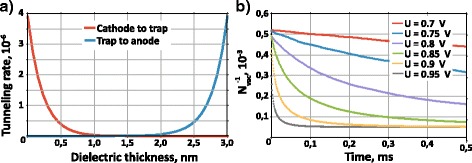
**a** Tunneling rates for 3 nm HfO_x_, obtained by WKB (Wentzel-Kramers-Brillouin) approximation. **b** Numerical modeling of stochastic trap generation defined for 3-nm HfO_x_

Such behavior can be explained in the following way. As it has been shown previously [23], the electroforming is an upsurge process with the exponential dependence of the forming time on the applied voltage. Therefore, there is no specific critical “forming” voltage and the electric field sufficient for the necessary oxygen vacancy generation in HfO_2_ [24] is already achieved during biasing of the device in a normal operation mode. The probability of an oxygen vacancy generation during the time interval d*t* depends on the local electric field force *F*_eq_ acting on the oxygen ion and is given by the formula [24]:

$$ P\left({F}_{\mathrm{eq}},T\right)=\frac{dt}{t_0} \exp \left(-\left({E}_a-\gamma {F}_{\mathrm{eq}}\right)/kT\right) $$

where *E*_*a*_ is the migration barrier height, *T* is the local temperature, 1/*t*_0_ is the characteristic vibration frequency of the oxygen ion, and γ is the fitting parameter representing local enhancement factor due to the electric field [25]. Neglecting variations of field components due to a filament growth and assuming that the model is efficiently one-dimensional, *F*_eq_ is inversely proportional to the oxide layer thickness: *F*_eq_ *~ V/L*. As a result, for as grown 3-nm-thick oxide layer, the efficient vacancy generation begins at voltages, which are comparable with consequent SET process voltage. Moreover, such ultrathin functional HfO_2_ layer also affects the tunneling probability across it. In fact, the regions where cathode-to-trap and trap-to-anode tunneling effectively occurs span through almost half of the layer cross section, which is confirmed by the calculation of the tunneling rates employing WKB approximation (Fig. 6a; the trap energy of the empty and filled O vacancies *E*_empty_ = 1.83 eV and *E*_full_ = 1.97 eV, respectively, below HfO_2_ conduction band). Consequently, every generated O vacancy trap contributes to the conductivity, so the resistance across HfO_2_ layer should be proportional to 1/*N*_vac_. The numerical modeling of the stochastic dynamics shows that for 3-nm-thick oxide layer, the process can generate enough oxygen vacancies to reduce structure resistance already at the bias U ~ 0.7 V (Fig. 6b; the parameters of simulation are 1/t_0_ = 10^13^ Hz and *γ* = 7.5, and the maximal number of oxygen vacancies is *N*_Vo_ = 40 × 40 × 3/0.25 = 1.84 ⋅ 10^4^, *E*_a_ = 1 eV).

**Fig. 7:**
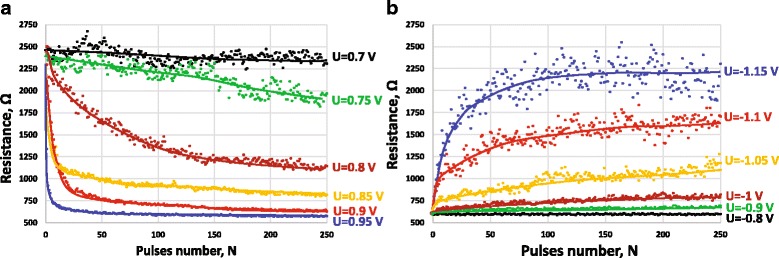
The resistance of 40 × 40 nm^2^ 4-nm-thick HfO_2_ device vs. biasing pulse number: **a** SET and **b** RESET transition

If the latter mechanism is true, the resistance state changes in our memristor devices should qualitative follow the stochastic dynamics of the trap generation shown in Fig. 6b. In order to verify this, we performed the measurements of the resistance response to the sequence of identical pulses (“pulse train” test, pulse width *τ*_*FWHM*_ = 1.5 μs, the resistance after each programming pulse measured at *U* = 0.1 V). The measured relationship of resistance vs. number of pulses *R = f(N)* for SET and RESET process and for different pulse amplitudes are displayed in Fig. 7a, b, respectively. It is seen from the plots that generally, the resistance monotonously changes following the voltage pulses. The obtained results allow us to conclude that the formed devices represent true functional memristive system controlled by charge [26], since the charge passed through the device is directly proportional to the number of pulses. Moreover, such behavior emulates the “long-term potentiation” and “long-term depression” functionalities. This property in biological synapses defines the synaptic plasticity—the ability of chemical synapses to change their strength, which is believed to be the major cellular mechanism underlying learning and memory [27].

It is worth noticing that the resistance changes monotonously only at a large scale, and there are pulse-to-pulse fluctuations, especially in the high resistance region. The latter property can be explained by the stochastic nature of the vacancy generation/annihilation process. Such behavior was also observed in biological synapses [28]. We believe that such characteristics do not pose significant problems for the implementations of machine learning algorithms due to their iterative nature. Sequential repetitions of learning iterations will eventually drive synaptic weights into required values. A particular impact on the learning performance will depend on a specific algorithm. It is likely that in some cases, such fluctuations, if kept within certain bounds, can provide regularization of the learning process or can be used to implement global optimization algorithm akin to simulated annealing.

Another learning mechanism of biological synapses is spike-timing-dependent plasticity (STDP) [29], which implies that the change of synaptic weight is a strong function of the timing between the pre- and post-neuron spikes. It is widely accepted that STDP is responsible for the Hebbian learning [30]. In order to emulate STDP functionality in the fabricated memristors, we use the previously proposed [31] and experimentally implemented [32] methodology. Briefly, the electrodes of the device are connected to two separate arbitrary waveform generators serving as pre- and post-synaptic neurons. The output voltage pulses emulate the shape of real neuron spikes [33]: the negative trapezoidal pulse (*τ*_FWHM_ = 2 μs with 1 μs edges) pulse with *U* = −0.6 ÷ −0.8 V amplitude (adjusted for each particular structure and generally different for pre- and post-synaptic neurons), followed by a long (*τ* = 1 ms) positive decaying triangular tail with the maximal amplitude *U* = 0.6 V.

**Fig. 8:**
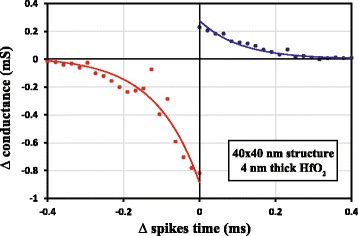
Asymmetric STDP function emulated in crossbar 40 × 40 nm^2^, 4-nm-thick HfO_2_ memristors

The relative change of the conductance Δ*G* as a function of the spikes’ delay time Δ*t* obtained from 4-nm-thick HfO_2_ 40 × 40 nm^2^ device is shown in Fig. 8. Both branches of asymmetric curves can be fitted with an exponential law as expected for this type of spike signal shape, thus representing STDP functionality. It is worthy to note that the observed STDP function of our crossbar memristive devices is similar to that displayed by biological synapses [29].

The emulated long-term potentiation/depression as well as STDP functionalities indicate the suitability of nanoscale TiN/HfO_2_/Pt memristor devices for the role of electronic synapses and thus they can be used for hardware implementation of hybrid CMOS/memristor neural networks (CMHNN). It should be noted, however, that the energy consumption in the reported memristor devices, which is currently ~1 ÷ 10 nJ per SET and RESET pulse resulting from the current level ~1 mA is far too large for the design of dense neural networks. The problem can be solved by employing precise current limiting using transistors in CMHNN during the switching of memristors in a crossbar matrix.

## Conclusions

In this work, we describe the electrical and synaptic properties of TiN/HfO_2_/Pt memristors with the lateral size down to 40 × 40 nm^2^ in crossbar geometry. The developed fabrication procedure was used to grow simple matrices of memristors with 3- to 5-nm-thick HfO_2_ functional layers exhibiting reversible resistive switching effect. Three-nanometer-thick devices are forming-free, with endurance up to 7 × 10^4^ cycles and *R*_On_/*R*_Off_ ~ 3 ÷ 20. Fabricated devices integrate current pulses exhibiting long-term potentiation and depression properties similar to that of biological synapses. Furthermore, by applying the voltage pulses emulating real biological spikes, the spike-timing-dependent plasticity functionality in 40 × 40 nm^2^ devices is demonstrated. Fabricated TiN/HfO_2_/Pt devices in crossbar geometry are promising candidates for hardware implementation of hybrid CMOS-neuron/memristor-synapse neural networks.

## Abbreviations

ALD: atomic layer deposition
CMHNN: hybrid CMOS/memristor neural network
CMOS: сomplementary metal-oxide-semiconductor
DC: direct current
STDP: spike-timing-dependent plasticity
